# Local Pain Collectives: A Novel Strategy for Improving Pain Knowledge, Pain Care and Community Connectedness in Rural Settings

**DOI:** 10.1111/ajr.70189

**Published:** 2026-04-22

**Authors:** Ashley R. Grant, Tanushka Alva, Mouli Baralaman, Shelley Barlow, Mieke Boerema, Charlotte Byrnes, Stuart Canavan, Jessica Fishburn, Kal Fried, Kate Johnson, Parivesh Kumar, Phil Ladlow, Alison Mattock, Abbie Norrish, Peter Roberts, Karina Savur, Sophie Shephard, Alex Stronach, Lauren Young, Emma L. Karran, Dianne Wilson, Carolyn Berryman, G. Lorimer Moseley

**Affiliations:** ^1^ Innovation, Implementation and Clinical Translation (IIMPACT) in Health Adelaide University Adelaide South Australia Australia; ^2^ Pain Revolution Adelaide University Adelaide South Australia Australia; ^3^ InBalance Launceston Tasmania Australia; ^4^ Truecare Physiotherapy Sale Australia; ^5^ Ballina Community Health Ballina Australia; ^6^ Physio Alive Aldinga Beach Australia; ^7^ Ocean Road Allied Health and Movement Anglesea Australia; ^8^ Kardinia Health Belmont Australia; ^9^ Regional Institute of Sport Camperdown Australia; ^10^ Gen Health Hamilton Hamilton Australia; ^11^ Rehabilitation Medicine Group Brighton Australia; ^12^ Kate Johnson Osteo Albury Australia; ^13^ Gippsland Lakes Complete Health Lakes Entrance Australia; ^14^ AllCare Physiotherapy Hobart Tasmania Australia; ^15^ South Coast Osteopathy Oak Flats Australia; ^16^ Peter Roberts Physio Adelaide Australia; ^17^ Melbourne Pregnancy and Pelvic Floor Physiotherapy Essendon North Australia; ^18^ School of Allied Health, Exercise and Sports Science Charles Sturt University Wagga Australia; ^19^ Medicine in Motion Health Group Kiama Australia; ^20^ Riverside Physiotherapy Launceston Tasmania Australia

**Keywords:** chronic pain, guideline implementation, isolation, meeting report, rural health

## Abstract

**Objective:**

To present key discussion points from a workshop where attendees reflected on personal and professional development achievements gained through their involvement in Pain Revolution's ‘Local Pain Collectives’ program.

**Design:**

Reflexive thematic analysis of notes written by workshop attendees and key points discussed during workshop activities.

**Setting:**

An in‐person workshop was held in Melbourne, Victoria.

**Participants:**

A cohort of Pain Revolution Local Pain Collective facilitators, their mentors and Pain Revolution's executive team.

**Results:**

Twenty‐eight meeting attendees (19 facilitators, six mentors and three executive team members). Facilitators reported gaining increased knowledge of contemporary pain science, greater confidence communicating with other healthcare providers and improved interactions with their patients. Further, they related that their local collective members reported similar improvements in their understanding of pain and their patient interactions. They described that their collectives enabled new professional connections, enhanced a sense of belonging to a community of like‐minded healthcare providers and increased collaborative pain care in their communities.

**Conclusions:**

The increased connection, sense of belonging to a community of like‐minded healthcare providers, and formation of collaborative care networks are all important program impacts given the lack of existing literature regarding interventions targeting professional isolation.

## Key Points

What Is Already Known on This Subject?

- Rural healthcare providers are often challenged by a lack of support and isolation.
- Rural Australians often lack access to specialised pain care services.

What This Paper Adds?

- The ‘Local Pain Collectives’ program supports rural healthcare providers to gather local healthcare providers together to discuss pain science education topics and to promote improved pain care.
- Rural healthcare providers who facilitated these collectives described gaining knowledge, confidence, skills, and a sense of belonging in their communities.
- This report offers the outline of an initiative that rural healthcare professionals could adapt for their own use to enable them to connect with other local providers through interdisciplinary learning. This report also offers evidence that rural healthcare professionals could use when seeking support to organise such programs given our summation that programs like this may improve retention of rural healthcare providers and facilitate the delivery of guideline‐recommended interdisciplinary pain care.

## 1 Introduction

Rural healthcare providers (HCP) experience unique opportunities, advantages, and challenges. They benefit from a wide scope of practice and a desirable small‐town lifestyle [1, 2, 3]. Doing more of what they are educated and competent to do presents increased opportunities for career progression and satisfaction, yet rural HCPs frequently describe common challenges including limited support, isolation and lack of access to professional development [2, 4, 5, 6, 7]. Each rural setting is unique, but it is common to lack access to desired healthcare services [7, 8] including specialised pain services [9, 10], making it difficult to implement guideline‐recommended interdisciplinary chronic pain care [11].

**FIGURE 1:**
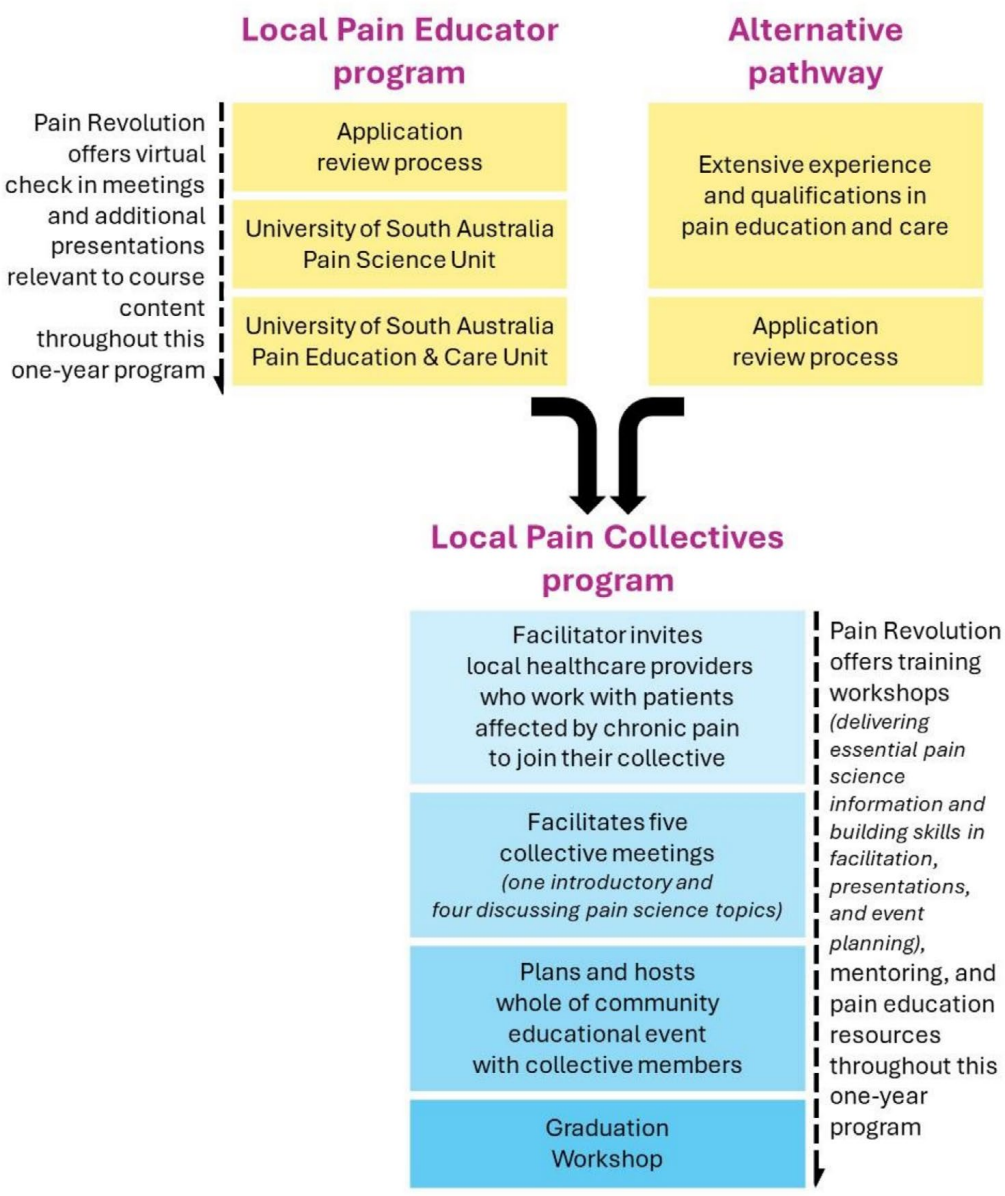
Local pain collectives program.

Pain Revolution is a not‐for‐profit that strives to build sustainable pain care in rural and regional areas [12]. Pain Revolution's initiatives include capacity building programs that endeavour to enhance rural HCPs pain‐specific understanding and skills. Rural HCPs apply to undertake these programs and are selected based on their capacity and motivation to promote improvements in pain care in their community. Development of these programs was informed by rural HCPs' reports that they lacked confidence in treating people with chronic pain, that they felt isolated in their clinical practice, and that referral networks were difficult to establish. To help address these challenges, Pain Revolution offers a two‐year program that integrates two initiatives—the ‘Local Pain Educator’ and ‘Local Pain Collective’ initiatives. The first year involves Local Pain Educator training via a Professional Certificate in Pain Science and Education offered online by the University of South Australia. HCPs who already have advanced knowledge and skills in pain can forego this first‐year certificate program. The second year involves individualised mentoring and a series of workshops and seminars during which candidates establish and become facilitators of a Local Pain Collective. Local Pain Collectives are interdisciplinary groups that include medical and health professionals from a given geographical area. With support from Pain Revolution, the Local Pain Collective facilitators organise a series of professional development and strategic networking sessions within their collective and then lead the Collective's delivery of one or more community‐facing pain education events. Figure 1 presents the Collective facilitators training journey.

At a graduation workshop for a cohort of Pain Revolution Collective facilitators, attendees engaged in a guided evaluative reflection on the program. The objective of this meeting report is to share common personal and professional development impacts described by this cohort. Highlighting the positive impacts of this type of program offers evidence regarding why it may be beneficial for rural HCPs to spread their knowledge with their local communities.

## 2 Methods

### 2.1 The Workshop

In February 2023, a cohort of Pain Revolution Collective facilitators and their mentors gathered in Melbourne to attend a graduation workshop to celebrate the completion of their one‐year program. Three Pain Revolution executive team members led the workshop, namely GLM [PhD, CEO and founder], AN [BPhysio, General Manager], and TA [MMed (PainMgt), Local Pain Educator Program Manager]. They each possessed existing relationships with attendees developed through the Collective program. At the start of the workshop, GLM informed attendees that ARG, who was not involved in the Collective program, was attending to record notes on group discussions for a potential meeting report manuscript, pending attendees approval.

The workshop began with a presentation on the evolution of Pain Revolution, followed by three group activities developed by Pain Revolution's executive team. The first activity focussed on impacts of the program, which will be the focus of this manuscript. Participants recorded their own achievements, the achievements they observed in their Collective members and within their communities. The second activity was ‘future dreaming’. Participants generated statements describing changes they wanted to see in their communities by 2028. The third activity sought to generate specific action participants could implement after the workshop to progress towards their visions for 2028. Participants recorded their responses on colour coded notes and organised them by activity on a wall. Small group discussions followed each activity, then all attendees discussed interesting or commonly identified points to deepen group understanding.

### 2.2 Data Analysis

We followed Braun and Clarke's six‐phase reflexive thematic analysis process [13] to identify patterns in expressed program achievements. At the workshop, author ARG recorded key discussion points during post‐activity group reflections to ensure topics highlighted as significant to the group were present in the final themes. After the workshop, ARG physically grouped participant notes into clusters of common content, organising into themes and subthemes, then entered these themes alongside all raw content into a Word document. The core research team [ARG, CB, DW, ELK, GLM] met to review the themes and prepared a one‐page summary document describing the key meeting discussion points. Authors ARG and AN reviewed the developed themes and revised the summary. In April 2023, all workshop attendees reviewed the one‐page summary to reach consensus that the final themes reflected workshop discussions. Using a Qualtrics [14] survey, all attendees were asked for their permission to publish a report of the meeting's discussions, to provide comments to be considered during manuscript preparation, and were invited to join the authorship team, including reviewing and editing this report. Once approval from all attendees was received, we generated this report with reference to the COREQ guidelines [15].

## 3 Results

Nineteen Collective facilitators, six mentors, and three Pain Revolution executive team members participated in the workshop. Demographic data for the 22 attendees who accepted the authorship invitation are presented in Table 1. Six attendees (two mentors, four facilitators) rejected the authorship invitation without providing reasons for rejection.

**TABLE 1:** Workshop attendees demographic data.

	Facilitators (*n* = 15)	Mentors (*n* = 4)	Executive team members (*n* = 3)	(*n* = 22)*
Sex assigned at birth				
Female	11 (73%)	2 (50%)	2 (67%)	15 (68%)
Male	4 (27%)	2 (50%)	1 (33%)	7 (32%)
Clinical profession				
Physiotherapist	10 (67%)	3 (75%)	3 (100%)	16 (72%)
Osteopath	3 (20%)	—	—	3 (14%)
Exercise Physiologist	1 (7%)	—	—	1 (5%)
General Practitioner	1 (7%)	—	—	1 (5%)
Sports & Exercise Physician	—	1 (25%)	—	1 (5%)
Highest degree earned				
Bachelor's degree	1 (7%)	1 (25%)	—	2 (9%)
Post‐graduate certificate	6 (40%)	—	1 (33%)	7 (32%)
Masters	7 (47%)	1 (25%)	1 (33%)	9 (41%)
PhD	1 (7%)	2 (50%)	1 (33%)	4 (18%)
State				
Victoria	7 (47%)	1 (25%)	—	8 (36%)
New South Wales	5 (33%)	—	—	5 (23%)
South Australia	—	3 (75%)	1 (33%)	4 (18%)
Tasmania	2 (13%)	—	1 (33%)	3 (14%)
Western Australia	1 (7%)	—	1 (33%)	2 (9%)
Rural classifications				
Metropolitan area	2 (13%)	4 (100%)	2 (67%)	8 (36%)
Regional centre	3 (20%)	—	1 (33%)	4 (18%)
Large rural town	4 (27%)	—	—	4 (18%)
Medium rural town	5 (33%)	—	—	5 (23%)
Small rural towns	1 (7%)	—	—	1 (5%)

* Missing data for six attendees who did not join authorship team.

We organised themes into three categories according to the focuses provided in activity one. These categories described the achievements obtained by (i) Collective facilitators; (ii) their Collective members; and (iii) their wider communities. Table 2 details themes and subthemes for each category, accompanied by illustrative quotes that portray the complexities and nuances in the data regarding how each theme and subtheme diversely manifested for individual meeting attendees.

**TABLE 2:** Themes, subthemes, and illustrative quotes.

**Level of impact**	
Theme	
Subtheme	Exemplar quote (s) from written sticky notes
**Facilitator achievements**	
Enhanced knowledge and skills	
Skills development‐problem solving, presentation, and technology management	“learnt how to design promotional materials.” “learnt about organising and catering for events.” “developing skills in: Zoom, social media, event organising, publicity, public speaking.” “improved skills in unpacking problems.”
Pain science knowledge + skills	“deepened knowledge and understanding of Essential Pain Facts.” “more knowledge on how to present + explain current best practice for people with chronic pain.” “professional growth, knowledge and skills.” “insights into the power of listening in education.”
Confidence	“improved confidence communicating with varied professions.” “confidence to back myself clinically + operate in a way consistent with my values.” “improved confidence communicating and facilitating ideas.”
Improved patient interactions	“being able to incorporate pain rev resources and concepts into the practice/service.” “able to change beliefs in some patients.” “better patient outcomes.”
Connection	
Being part of something	“feeling part of something BIG.” “feeling part of community that supports.”
Local networking	“finding allies in the community.” “community networking with local allied health professionals and GPs.” “increased referral and collaborative network.” “developed stronger professional relationships with local HCP.”
Likeminded providers	“given me access to a like‐minded evolving community: belonging, not alone.” “finding ‘my people’.”
Support	“feeling part of community that supports me.”
Personal growth	
Passion	“revived my passion for my career as a physio and my role in improving access to health care in my community.”
Inspiration	“feeling inspired and motivated.” “awareness of new/different possibilities for future growth.”
Values and personal empowerment	“feeling empowered to advocate and embody change.” “fuelling up to keep going.”
**Local pain collective member perceived achievements**	
Networking	
Attracting more committed members	“momentum building–organically built word of mouth.” “engagement, not just attendance.”
Connection	“not feeling isolated in community.” “meeting with the community and seeing who was like‐minded.”
Interdisciplinary and cross system communication	“open up communication between public and private practitioners.” “variance in professions–chiro, pharmacy, podiatry, wound nurse.”
Referral networks	“GP referrals to pain ed/chronic pain management.” “referral process increased.”
Patient impact	“positive outcomes for clients.” “consistent application of EPFs (Essential Pain Facts) when managing patients across local collective.”
Supportive resource access	“participants shared resources and management techniques for chronic pain patients.” “Collective = resource hub.”
Community appreciation	“acknowledgement/thank you from HCPs especially interns/new grads/to start LPC in country.” “thank you from community to share knowledge with them + request to organise more events to help.”
Spread of pain science education	“allow community practitioners to share their skillset and knowledge.” “awareness of Pain Revolution + research in the space increased ++.” “‘converted some clinicians to get ‘curious’‐participants from group who have gone on and spread the concepts into their networks.”
**Wider community perceived achievements**	
Enhanced local awareness	
Of contemporary pain science	“increased awareness in community of alternative methods of pain management.”
Of local pain care options	“increased awareness in community of who ‘pain champions’ in local area are.”
Enhanced connection	
New referral networks	“connect to ‘safe’ on topic health providers to refer to or to ask who they recommend.”
Network of providers sharing same messaging to patients	“‘common language’‐breaking down of silos, shared understanding, inclusion, shared goals.” “great spread of different people–disciplines, private/public.”
Support in local community	“feeling supported/not feeling burnt out.” “community feeling able to discuss how difficult this work is and get support.”
Positive patient impact	“patients outcomes improving.” “patients educating family/other members.”

Local Pain Collective facilitator achievements—Participants wrote 80 notes, reporting 136 individual achievements. Facilitators experienced growth in their knowledge of contemporary pain science, their confidence communicating with patients and other HCPs, and improvements in their patient interactions. They reported developing problem solving, presentation and technology management skills. Further, facilitators described gaining connections and a sense of belonging and support.

Local Pain Collective member achievements—Twenty‐eight notes described 56 Collective member achievements. Facilitators perceived their respective collective members improved their understanding of pain, valued their increased access to resources, and believed the quality of their patient interactions had improved. Further, they observed the formation of new connections, including interdisciplinary and cross‐system level communication and new referral networks.

Wider community achievements—Twenty‐eight notes presented 49 community‐wide achievements. Facilitators observed increased community awareness of contemporary pain science and of local pain care options. This was evident by consumers seeking care from Collective members after community education events, Collective members receiving referrals from HCPs who attended community events, and new patients asking informed questions, including probing how they could manage pain without medication. Additionally, facilitators described improvements in patient outcomes, increased collaborative pain care, and consistency in pain messaging across multiple care providers.

## 4 Discussion

We reported achievements described by rural HCPs who engaged in a training program that supported them to share their pain knowledge with their communities. Attendees reported that the Local Pain Collectives program increased their pain knowledge and clinical confidence, improved their patient care, and allowed them to feel more connected to and supported by other HCPs. Attendees described how this program appeared to have similar positive impacts for their Collective members and their communities.

Outdated approaches in pain care have been suggested to be the norm in rural communities and this can negatively affect rural pain care providers' provision of contemporary pain science informed care [16]. The Collectives program enables rural pain care providers to develop relationships with others providing care in this way and facing similar challenges; hence, a common theme at all three levels of impact is gained connection, support and sense of belonging. These are important program impacts because isolation and lack of support are common challenges affecting rural HCPs [2, 4, 5, 6, 7], and there is limited literature reporting on interventions to overcome these challenges [17]. Providers who experience limited support report feeling stressed, isolated, and lacking confidence in their skillset; and this may lead providers to leave rural clinical practice [4, 18]. Alternatively, networking, supportive relationships, social connection and creating a sense of belonging—all suggested benefits of this program—have been linked to retention of health workforce in rural settings [19, 20].

The Collectives reportedly enhanced interdisciplinary communication pathways and referral networks with the potential for positive impacts on the implementation of guideline‐recommended interdisciplinary pain care [11]. A further anticipated benefit of the program is the increased consistency of pain messaging across the providers in these communities. This has the potential to facilitate knowledge gain and improve patient experiences of care [21]. Participants perspectives strongly suggest that the impetus for developing the LPC program was successfully targeted. Thus, the findings of this meeting report suggest that regional or rural HCPs who lack confidence, experience isolation in their clinical practice, or find it difficult to establish referral networks within their region may be able to set up their own version of these Collectives to help achieve these same gains.

## 5 Strengths and Limitations

A strength of this report is that Collective facilitators came from diverse rural settings, which supports the generalisability of program impacts. Meaningful engagement of workshop attendees at all stages of manuscript production ensured that interpretations made during thematic analysis represented the workshop discussions as accurately as possible. We acknowledge, however, that a limitation of this report is that the workshop focused only on positive program impacts and did not explore challenges faced or things that did not work. Further, many participants described changes in their behaviours (e.g., networking, referral patterns) in response to the programs, however we acknowledge that due to relying on self‐report data we are unable to be certain whether these changes actually occurred. Certainly, it appears from subjective reports (see Table 2) that there were many positive impacts on HCPs sense of connectedness, support and belonging, but we did not complement our qualitative approach with quantitative analysis of connection (e.g., via referral numbers, validated questionnaires or well‐being markers). Notwithstanding, our primary focus was to understand the perspectives of the LPC facilitators themselves.

## 6 Conclusion

This meeting report presents achievements reported by rural HCPs with advanced pain skills and knowledge who were supported to spread pain knowledge with their communities. Supporting a rural HCP to form a local collective of interest‐holders seems to enable increased knowledge and confidence, generate referral networks, connections, a sense of belonging, and improve patient care. These impacts are important to highlight because similar programs could improve retention of HCPs in rural communities, facilitate implementation of guideline‐recommended care and improve patient outcomes and experiences of care.

## Author Contributions

**Shelley Barlow:** data curation, writing – review and editing. **Mouli Baralaman:** writing – review and editing, data curation. **Kate Johnson:** data curation, writing – review and editing. **Ashley R. Grant:** conceptualization, investigation, writing – original draft, methodology, formal analysis, project administration. **Kal Fried:** writing – review and editing, data curation. **Alison Mattock:** data curation, writing – review and editing. **Stuart Canavan:** writing – review and editing, data curation. **Jessica Fishburn:** data curation, writing – review and editing. **Charlotte Byrnes:** data curation, writing – review and editing. **Phil Ladlow:** data curation, writing – review and editing. **Sophie Shephard:** writing – review and editing, data curation. **Peter Roberts:** writing – review and editing, data curation. **Tanushka Alva:** resources, project administration, writing – review and editing, methodology. **Emma L. Karran:** writing – review and editing, supervision. **Alex Stronach:** writing – review and editing, data curation. **Lauren Young:** writing – review and editing, data curation. **G. Lorimer Moseley:** writing – review and editing, funding acquisition, data curation, supervision. **Carolyn Berryman:** writing – review and editing, data curation, supervision. **Karina Savur:** data curation, writing – review and editing. **Abbie Norrish:** data curation, resources, project administration, writing – review and editing, methodology.

## Funding

GLM, ELK and CB are supported by a Leadership Investigator Grant from the National Health & Medical Research Council of Australia ID 1178444 (held by GLM). ARG is supported by a post‐graduate research scholarship from the Rural Doctors Workforce Agency (RDWA) of South Australia and was supported for travel and accommodation for this meeting from NHMRC Grant ID 1178444 (held by GLM). CB is supported by an MRFF Multidisciplinary Models of Primary Care Grant (MRF2040282). TA and LW received funding from the Tasmanian Public Health Network (PHN). PK received funding from the Tasmanian Department of Health. An abstract of this work will support an oral presentation that will be given at the South Australian Rural Health and Education Conference in the Riverland of South Australia in August.

## Conflicts of Interest

G.L.M. has received support from Reality Health, ConnectHealth UK, AIA Australia, Institutes of Health, California, Kaiser Permanente, Workers Compensation Boards in Australia, Europe and North America, and various sporting organisations internationally. G.L.M. receives royalties for several books on pain and speakers fees for talks on pain, pain education, physiotherapy, and rehabilitation. G.L.M. has received support from professional societies, rehabilitation companies and governments for attendance at scientific meetings and departmental briefings. G.L.M. is founder and unpaid CEO of Pain Revolution; A.N., T.A. and D.W. are paid by Pain Revolution. E.L.K. has received support from professional societies for attendance at scientific meetings. C.H., J.F., P.L. and A.N. have received payment for delivering lectures on pain education. K.F. has shares in ‘Brain Changer’, a digital therapeutic intervention for chronic pain. A.M. has fees for lectures on pain education from Grand Pacific Health. A.N. received financial and professional development support from Pain Revolution. P.R. receives lecturing fees for lectures and professional development courses on pain, and travel support from the Australian Physiotherapy Association. C.B. has received speaker fees for lectures on pain and rehabilitation, received support from ReturnToWorkSA, Kaiser Permanente and from professional societies for attendance at scientific meetings. M.B., S.B., M.B., S.C., K.J., A.S., A.R.G., and D.W. declare no conflicts of interest.

## Data Availability

Research data are not shared.
